# The underestimated role of temperature–oxygen relationship in large‐scale studies on size‐to‐temperature response

**DOI:** 10.1002/ece3.3263

**Published:** 2017-08-11

**Authors:** Aleksandra Walczyńska, Łukasz Sobczyk

**Affiliations:** ^1^ Institute of Environmental Sciences Jagiellonian University Krakow Poland

**Keywords:** body size, diatoms, multicollinearity, nutrition, oxygen, temperature, temperature‐size rule, thermal preferences

## Abstract

The observation that ectotherm size decreases with increasing temperature (temperature‐size rule; TSR) has been widely supported. This phenomenon intrigues researchers because neither its adaptive role nor the conditions under which it is realized are well defined. In light of recent theoretical and empirical studies, oxygen availability is an important candidate for understanding the adaptive role behind TSR. However, this hypothesis is still undervalued in TSR studies at the geographical level. We reanalyzed previously published data about the TSR pattern in diatoms sampled from Icelandic geothermal streams, which concluded that diatoms were an exception to the TSR. Our goal was to incorporate oxygen as a factor in the analysis and to examine whether this approach would change the results. Specifically, we expected that the strength of size response to cold temperatures would be different than the strength of response to hot temperatures, where the oxygen limitation is strongest. By conducting a regression analysis for size response at the community level, we found that diatoms from cold, well‐oxygenated streams showed no size‐to‐temperature response, those from intermediate temperature and oxygen conditions showed reverse TSR, and diatoms from warm, poorly oxygenated streams showed significant TSR. We also distinguished the roles of oxygen and nutrition in TSR. Oxygen is a driving factor, while nutrition is an important factor that should be controlled for. Our results show that if the geographical or global patterns of TSR are to be understood, oxygen should be included in the studies. This argument is important especially for predicting the size response of ectotherms facing climate warming.

## 1 INTRODUCTION

The temperature‐size rule (TSR) has been the subject of intensive scientific debate since the classical paper by Atkinson (1994) who found, based on a meta‐analysis of empirical data, that over 80% of ectotherms adhere to this rule. However, the possible adaptive role and operational range of TSR are not recognized. In regard to the former, there are several hypotheses about how such a plastic pattern could evolve (Angilletta & Dunham, 2003; Angilletta, Steury, & Sears, 2004). One of the most promising suggestions states that the TSR evolved to optimize the temperature‐dependent oxygen demand and supply. This idea was conceptualized in two hypotheses, the MASROS (Maintaining Aerobic Scope by Regulating Oxygen Supply; Atkinson, Morley, & Hughes, 2006) and OCLTT (Oxygen‐ and Capacity‐Limited Thermal Tolerance; Poertner, 2010). According to both hypotheses, the steeper increase of oxygen demands than of oxygen supply with increasing temperature reduces the range of possible aerobic metabolism (=aerobic scope). The ectotherms apply certain performance to prevent this damaging process. The MASROS hypothesis refers directly to temperature‐dependent size response to such oxygen limitations; the reduction in size increases the diffusive surface relative to size and, in consequence, improves the oxygen consumption (Atkinson et al., 2006).

This prediction has sound empirical support, as benthic amphipods were found to be larger with increased oxygen content in water (Chapelle & Peck, 1999; but see also Makarieva, Gorshkov, & Li, 2005; and Verberk, Bilton, Calosi, & Spicer, 2011), *Drosophila melanogaster* was found to be smaller in hypoxia conditions, and this effect was enhanced at high temperatures (Frazier, Woods, & Harrison, 2001), smaller *Lecane inermis* rotifers laid more eggs, which indicates higher fitness, than larger individuals at high temperature/low oxygen conditions (Walczyńska, Labecka, Sobczyk, Czarnoleski, & Kozłowski, 2015), while the rotifer *Keratella cochlearis* increased with decreasing temperature in its natural habitat, although this pattern was constrained in poorly oxygenated water (Czarnoleski, Ejsmont‐Karabin, Angilletta, & Kozlowski, 2015).

There are also a number of documented exceptions to TSR. One such report is by Adams et al. (2013). The authors examined an extensive dataset of diatoms from fourteen geothermally heated freshwater streams in Iceland that differed in temperature by almost 20°C. Based on the temperature‐size relationship of the 31 species found, they determined that about half of them were following the TSR across all the streams, while the other half were not. This justified the general conclusion of the article that diatoms may be an important exception to TSR. Such a claim is potentially of great importance to the issue of global climate change, and it directly contradicts the predictions by Daufresne, Lengfellner, and Sommer (2009), among others, who demonstrated that a decrease in size of aquatic organisms is one of the most important ecological responses expected as a consequence of global warming.

Other published results focused on the temperature‐size response in diatoms, from studies conducted in field, show both findings, with reports for (Peter & Sommer, 2012; Svensson, Norberg, & Snoeijs, 2014; Winder, Reuter, & Schladow, 2009) and against the TSR response (Vanden Byllaardt & Cyr, 2011). Among them, Svensson et al. (2014) examined the relationship between diatom size and three environmental traits: temperature, salinity, and nutrient supply. The study was performed in the Forsmark area of the Baltic Sea. The results identified 405 diatom species that exhibited temperature‐size responses in accordance with TSR, which contradicts the conclusion of Adams et al. (2013). The authors suggest that the discrepancy between these two reports may be caused by the different temperature‐dependent nutrient supply in the two studies. In his classical paper, Atkinson (1994) formulated the TSR under conditions not limited by food. Indeed, the role of nutrition in TSR performance was previously reported in the studies on rotifers (Galindo, Guisante, & Toja, 1993; Kiełbasa, Walczyńska, Fiałkowska, Pajdak‐Stós, & Kozłowski, 2014; Wojewodzic, Rachamim, Andersen, Leinaas, & Hessen, 2011) and caterpillars (Lee, Jang, Ravzanaadii, & Rho, 2015). However, we suggest that while nutrition affects the TSR pattern, it is not its main driver (we discuss this point below). Our prediction is that a crucial factor, not taken into account in any of the diatom reports mentioned above, is the discrepancy between the temperature‐dependent oxygen demands and supply. The strong natural negative correlation between temperature and oxygen (Denny, 1993; Garcia & Gordon, 1992; Wetzel, 2001) means that TSR may be considered as being responsive with regard to temperature but anticipatory with regard to oxygen, possibly because mechanisms of response to temperature are better developed in living organisms than those of response to oxygen, as was previously suggested by Walczyńska et al. (2015).

In this study, we extensively investigated the data collected by Adams et al. (2013) to determine if the examined streams differed in regards to the general physical and trophic conditions and whether these potential differences affected the diatom temperature‐size response. The argument behind this approach is that the oxygen conditions were monitored in Adams et al. (2013) along with other physical factors, and including the oxygen conditions in the analyzes may shed new light on the diatom size differences across streams of different temperatures. We applied a multivariate analysis to account for the possible differences between streams. Our hypothesis is that the strength of the TSR response in diatoms differs in response to the oxygen conditions in the investigated streams. Specifically, we expect that the TSR is stronger at higher thermal ranges because these are the stressful conditions in regard to oxygen availability.

## 2 METHODS

### 2.1 Analysis of differences in characteristics among the streams studied

The originally studied area consists of fourteen streams in the Hengill region of Iceland, the water of which is differently altered by geothermal warming and ranges from 5 to 25°C (Adams et al., 2013). These streams were already the subject of research on different aspects of the effects of temperature: community structure and trophic interactions (Woodward et al., 2010), primary production (Gudmundsdottir et al., 2011), and individual *vs*. ecosystem‐level response (O'Gorman et al., 2012). The temperature was measured in the streams in August 2008, along with diatom sampling (Adams et al., 2013) and it was recorded on the same day in all streams (Woodward et al., 2010). To determine if the streams grouped according to specific conditions, we conducted a PCA analysis with a correlation matrix (CANOCO 5; Ter Braak & Šmilauer, 2012) using all the physico‐chemical parameters collected in the supplementary materials (table S1) of Adams et al. (2013). In a second step, we removed temperature and oxygen (the factors of our main interest) from the analysis to check if the other parameters differentially affected the stream aggregation. We also checked the correlation matrix (Pearson's correlation coefficient) of all the abiotic parameters (STATISTICA 10, Statsoft, 2014).

### 2.2 Analysis of diatom size response to temperature within stream groups

For this analysis, we used the species database from the Supplementary Materials in Adams et al. (2013; table S10), containing 37 diatom species analyzed for body size in an original article. The product of length and width (original values provided by the authors) was used as an approximate size measure (in the original work, the authors also estimated diatom area, but they used a genus‐specific standard geometric shape in their calculations). We checked the diatom temperature‐size relationship of each stream group, arbitrarily separated according to the PCA plot, with a linear regression analysis (data analysis using one statistical model with stream group and species as model factors was not possible because of the uneven distribution of species; only six species from an original species database were present in all three stream groups, and only one of them was present in all streams). Additionally, for each species within a stream group we estimated a coefficient of regression for the size‐temperature relationship and we conducted the effect‐size analyzes of grand mean for *r*, using a meta‐analysis tool (OpenMEE software, Wallace et al., 2016), for each stream group separately. We used a fixed‐effect model for Fisher's Z‐transformed effect size.

## 3 RESULTS

### 3.1 Stream differences

**Figure 1:**
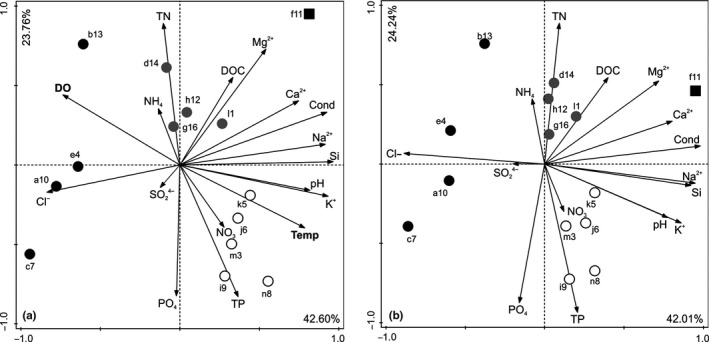
The PCA results illustrating the streams grouped according to the physico‐chemical characteristics, with temperature and oxygen included (a) or excluded (b) from the analyses. The following stream groups were selected: G1— represents cold, well‐oxygenated streams (black circles), G2—moderate temperature and oxygen, high nutritional value (gray circles), and G3—warm, poorly oxygenated streams (white circles). A black square indicates one stream that considerably differed in conditions from all other streams, and was therefore excluded from further analyses. Percentages of amount of variance explained by two axes are provided. Temp, temperature; Cond, conductivity; DOC, dissolved organic carbon; DO, dissolved oxygen; TN, total nitrogen; and TP, total phosphorous

The PCA analysis explained 42.6% of variation in axis 1 and 23.7% of variation in axis 2 and showed a clear pattern of streams grouped according to their physico‐chemical parameters (Figure 1). The loadings for all parameters included in the analysis are provided in Appendix S1. In addition, these aggregations were unchanged whether temperature and oxygen were included in the analysis or not and the percentage of variance explained by two axes was very similar (Figure 1). The correlation analysis showed that temperature was most strongly negatively related with dissolved oxygen (DO) and positively with pH and a few nutrient parameters, including NO_3_, total P, K, and Si (Appendix S2).

**Figure 2:**
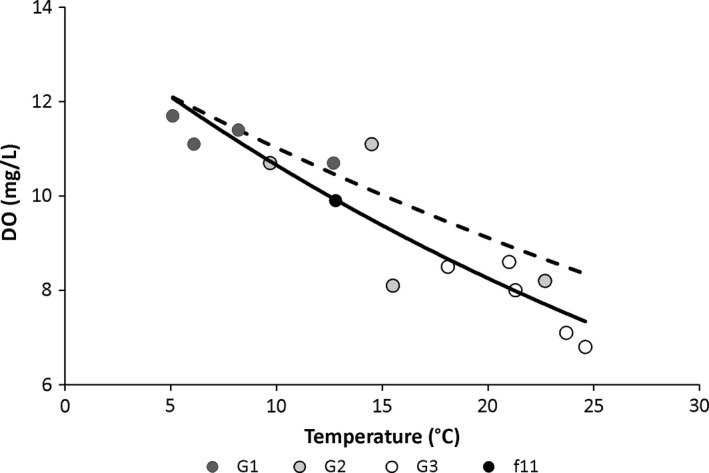
The temperature–oxygen relationship across the investigated streams (exponential estimation; continuous line). Dashed line shows the temperature–oxygen relationship for fully saturated water, estimated from data provided by Wetzel (2001). Data are differentiated by stream groups that were demarcated according to the PCA analysis (G1‐G3 and additional stream f11). G1—represents cold, well‐oxygenated streams, G2—streams with intermediate temperature and oxygen values, and G3—warm and poorly oxygenated streams. DO, dissolved oxygen

According to this pattern, we selected three groups of streams: G1 (cold, well‐oxygenated), G2 (moderate values of temperature and oxygen, but high values of nutrients), and G3 (warm, poorly oxygenated; Figure 1). One stream, f11, differed considerably from the other streams due to the exceptionally high nutrient concentrations (Mg^2+^, Ca^2+^ and DOC; Figure 1) and was excluded from further analyses. The temperature ranges of the three stream groups were 5.1–12.7°C for G1, 9.7–22.7°C for G2, and 18.1–24.6°C for G3. The oxygen content in the water was strongly and significantly negatively correlated with stream temperature (*r* ^*2* ^= .85, *r* = −.92, *p* < .0001) across the entire dataset (Figure 2). The comparison with curve illustrating the oxygen saturation decrease with increasing temperature for fully saturated water, estimated based on data from Wetzel (2001), shows that oxygen availability decrease with increasing stream temperature is more severe than caused by oxygen solubility only (Figure 2).

### 3.2 Species sizes

**Figure 3:**
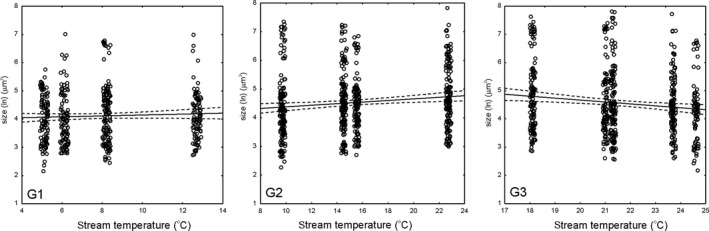
Linear regression of diatom size (μm^2^) on stream temperature (^°^C) with confidence intervals. The points were jittered on the *x*‐axis to improve visibility. G1 represents cold, well‐oxygenated streams, G2—streams with intermediate temperature and oxygen values, and G3—warm and poorly oxygenated streams

**Figure 4:**
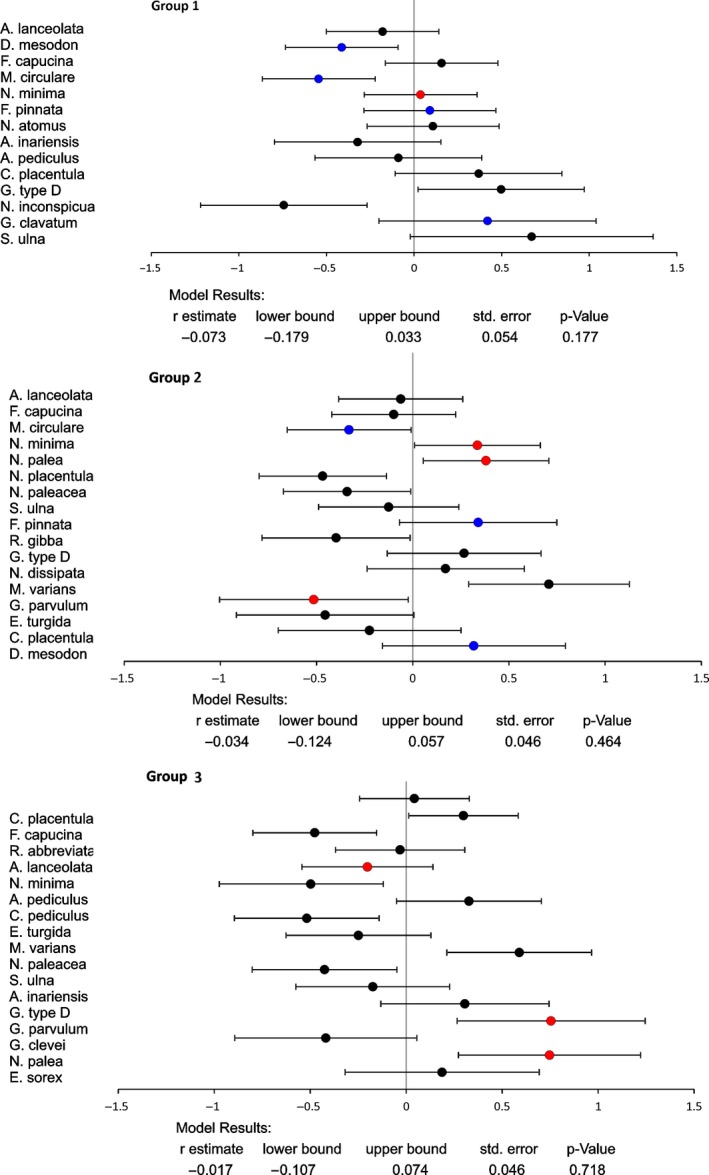
The effect‐size analyses (grand mean estimation of *r*) of the regression coefficients of the size‐temperature relationship, for each stream group separately (r‐value ±95% CI). G1 represents cold, well‐oxygenated streams, G2—streams with intermediate temperature and oxygen values, and G3—warm and poorly oxygenated streams. The species preferring cold OR high oxygen levels are marked in blue, and species preferring warm OR low oxygen levels are marked in red (information kindly provided by Agata Z. Wojtal). The preferences of other species are moderate or unknown. Species in each group are sorted from the highest to the lowest sample size value.

We filtered the original diatom database by removing (i) five species that occurred only in one stream, (ii) two species with only one individual measured in the database, and (iii) species that occurred only in one stream within a stream group as defined in the PCA analysis. This included 11 species from G1, 13 species from G2, and eight species from G3 (Appendix S3). The remaining number of species included in analysis, in respective groups, was 14, 17, and 17, and most of the species (but not all) were represented by ten individuals measured. The within‐group results showed no temperature‐size relationship in G1 (*p* = .27, *R* ^2 ^= 0.003, *b *=* *0.018 ± 0.016 *SE*, where *b* denotes slope), a significantly positive relationship in G2 (*p* = .003, *R* ^2 ^= 0.015, *b *=* *0.028 ± 0.009 *SE*), and a significantly negative relationship in G3 (*p* = .002, *R* ^2 ^= 0.017, *b *=* *0.068 ± 0.022 *SE*) (Figure 3). The effect‐size analyzes showed no significant grand mean estimate in any group, caused mostly by the species‐specific differences in response (Figure 4).

## 4 DISCUSSION

The results of our study confirm the predictions that the temperature–oxygen relationship is crucial in TSR performance in diatoms from the thirteen Icelandic streams. The PCA analysis included all the physico‐chemical parameters collected and grouped the streams into three clear clusters (Figure 1) distinguished by their temperature–oxygen conditions (these two factors were highly negatively correlated; Figure 2). Diatoms from cold streams differed in their TSR response to those from warm streams, where the discrepancy between temperature‐dependent oxygen demand and temperature‐dependent oxygen supply is the greatest (Verberk et al., 2011). The increase of the discrepancy between oxygen demands and supply with increasing temperature refers to natural conditions; under stressful conditions the size‐to‐temperature response differs from the TSR because of the physiological constraints for organisms (Atkinson, Ciotti, & Montagnes, 2003; Walczyńska, Kiełbasa, & Sobczyk, 2016). Thought the temperature data were collected only once, and therefore, some important information on fluctuations is missing, yet, based on other studies, aquatic ectotherms adjust their size seasonally in response to temperature changes in time (Horne, Hirst, Atkinson, Neves, & Kiorboe, 2016; Kiełbasa et al., 2014), which makes the link between size as response to point measure of temperature reliable.

The important point here is that the diatoms from the original study were collected in their natural habitat and the temperatures in the streams were not stressful to them, meaning that they did not impair their physiological processes. According to our predictions, the TSR response was the strongest in diatoms from the warm stream group. The slope of our estimation of the relationship between dissolved oxygen and temperature tended to differ from the respective slope for fully saturated water (package SMATR in R; *p* = .064), meaning that diatoms from hottest streams could potentially experience lower oxygen availability than resulted just from the poorer oxygen solubility (Figure 2), the fact most probably caused by intensive activity of organisms consuming oxygen. Diatoms from the cold stream group showed no relationship, while those from moderate conditions showed the reverse pattern, most possibly driven by nutrition conditions.

The role of nutritional conditions in the temperature‐size response confirmed the general predictions by Atkinson (1994) and the suggestion regarding the diatom special case by Svensson et al. (2014). The importance of nutrition is best illustrated by stream f11, which was excluded from our analysis because of its considerable difference from the other streams (Figure 1). In this stream, temperature, and oxygen were intermediate, but nutrient levels were very high. Accordingly, all the diatoms from this stream were exceptionally large (data not shown). However, it is worth mentioning that the effect of temperature‐dependent nutritional conditions is fundamentally different from the effect of temperature‐dependent oxygen availability in regard to TSR. Now, we would like to present our point of view to this issue.

First, oxygen concentration in water is usually highly negatively correlated with temperature (e.g., Denny, 1993; Garcia & Gordon, 1992), while nutrient concentration generally increases with increasing temperature, which was the case in the study by Adams et al. (2013) (Table S1 therein). Because the TSR is a plastic response to temperature‐dependent oxygen availability, ectotherms should follow the pattern: Lower oxygen availability and higher temperatures lead to smaller body sizes, while higher nutritional values promote an increased body size. The oxygen concentration at ambient temperatures drives the TSR response, while nutritional conditions affect its strength. In other words, nutrition itself is not responsible for the TSR because it is difficult to imagine a mechanism in which body size decreases with increasing temperature in response to increased nutrient levels. The stronger effect of nutrition than of oxygen may be observed when temperature‐dependence of the former is steeper than that of the latter, what in our case might happen in Group 2, representing intermediate values of temperature and oxygen conditions.

The effect‐size analyses revealed another important factor affecting the TSR pattern in interspecific comparisons; the individual characteristics of species. In our data reanalysis, within each group there were species that exhibited significant decreases (TSR) or increases in size (reverse TSR) with increased temperatures (Figure 4), causing a general pattern of not significant mean effect size in any stream group. In our opinion, these results were mostly caused by the species‐specific thermal and oxygen preferences. In the dataset from Adams et al. (2013), three species preferred high oxygen levels (*Diatoma mesodon*,* Fragilaria pinnata,* and *Gomphonema clavatum*) and three preferred low oxygen levels (*Gomphonema parvulum*,* Navicula minima,* and *Nitzschia palea*), while the rest preferred intermediate oxygen values or the information on oxygen preferences were unknown (A. Z. Wojtal, personal data). We included the information on these preferences in Figure 4. Additionally, we treated one species preferring low temperature, *Meridion circulare*, as those preferring high oxygen (the information on known thermal preference for species from our dataset was much scarcer than for oxygen preferences). Accordingly, the cold/high oxygen preferring species were absent in streams from Group 3, while hot/low oxygen preferring species were underrepresented in streams in Group 1 (Figure 4). It was recently proposed that ectotherms exhibit TSR only within an optimal, species‐specific thermal range. The blind choice of temperature values in comparing the TSR in species with different thermal preferences (generalist *vs*. specialist or cold‐ *vs*. warm‐preferring) may cause the wrong conclusions and the existing TSR might not be detected (Walczyńska et al., 2016). In the case of this study, the effect of different preferences is not distinguishable from the effect of sample size (the reliability of each effect‐size estimation increases with increasing sample size) and the influence of the strategy associated with other species characteristic, such as life form, which in the case of diatoms may be motile/attached/floating and solitary/colonial. These are the reasons why our regression analysis, where the individuals from all species were pooled in order to save the degrees of freedom and therefore to increase the statistical power of the test, is more reliable regarding the general size‐temperature response, while effect‐size analyses show an illustration on why the regression estimation was weak.

We were not able to account for interspecific size response to temperature in the system studied because species composition differed between stream groups. It means that our results should be treated as a community‐level, rather than species‐level response. Actually, the species substitution was also examined by Adams et al. (2013). The authors concluded on no response to temperature neither at population nor at community level. Therefore, another interesting contribution of ours is that changes in species composition are observed when both temperature and oxygen gradients are taken into account. Some additional information regarding the occurrence in stream group and species preferences (if known) are discussed above and presented in Figure 4. The new and important insight into this issue would be possible if species characteristics were known; a combination of temperature and oxygen preferences, species size, shape, and life form all found a general species strategy to deal with suboptimal temperature and oxygen conditions.

Adams et al. (2013) correlated diatom body size with other abiotic variables than temperature, including pH, Si, K, NO_3_, NH_4_, total N, PO_4_, and total P. The authors concluded that temperature was not exceptional in its effect on diatom body size and that it was a less important driver of size variation than other factors, particularly pH, Si, and K. Interestingly, the authors' and our correlation analysis of Adams et al. (2013) abiotic data showed that all the three parameters, pH, Si, and K, were strongly related to temperature (Appendix S2). The problem with such an analysis is multicollinearity because it is difficult to disentangle the effects of two correlated variables (Quinn & Keough, 2005) and an approach to reliably distinguish such effects is not a trivial matter. Maranon, Cermeno, Latasa, and Tadonleke (2012) made attempts to disentangle temperature and resource effects on the size of phytoplankton in the ocean. Based on their analysis, the authors concluded that the role of temperature in shaping the size structure was negligible in their study system. However, Lopez‐Urrutia and Moran (2015) questioned their method and after reanalyzing the data, the authors found that temperature did explain the high variance in the size distribution of oceanic phytoplankton. Additionally, in the study on seasonal body size response to temperature and food concentration in copepods, Horne et al. (2016) disentangled these two factors and concluded that though they both mattered in body size response, yet temperature was its driver.

Of course, similar arguments apply to the temperature–oxygen relationship. The empirical solution is either to separate temperature from oxygen experimentally, as was previously conducted by Walczyńska et al. (2015) who reported on the adaptive role of oxygen and TSR in the case of a rotifer, and by Hoefnagel and Verberk (2015) who adopted this method to their study on the TSR in a crustacean, or by artificially controlling oxygen regardless of temperature. For example, such conditions exist in an activated sludge system. Studying the TSR in this system, Kiełbasa et al. (2014) showed that two rotifer species responded in size to seasonally changing temperatures in accordance with the TSR, and at least one of them considerably responded to seasonally differing oxygen concentration (a third factor, the external mortality, affected the general pattern). In this study, we chose an indirect method of dividing the study system into distinct assemblages of differing temperature–oxygen conditions.

Our results emphasize three important points, which in our opinion should be taken into account in studies on the geographical patterns of TSR: (i) The size response should be examined in regard to the temperature–oxygen relationship, (ii) the nutritional level should be controlled as a factor that shapes the strength of the TSR response, and (iii) a method for distinguishing the highly correlated effects of temperature, oxygen and nutrition should be carefully designed.

## CONFLICT OF INTEREST

None declared.

## DATA ACCESSIBILITY

This study is a reanalysis of already published and available data (Adams et al., 2013).
