# Supplementary material for: The underestimated role of temperature–oxygen relationship in large‐scale studies on size‐to‐temperature response
Source: Ecol Evol. 2017 Aug 11;7(18):7434–41. doi: 10.1002/ece3.3263 (PMC5606864; doi:10.1002/ece3.3263)
Supplement: Supplementary file 1 [file ECE3-7-7434-s001.pdf]

	axis 1	axis 2
Temp	0.79	-0.40
pH	0.82	-0.16
Cond	0.92	0.33
DOC	0.33	0.55
DO	-0.74	0.44
NH4	-0.14	0.35
NO3	0.28	-0.39
TN	-0.10	0.89
PO4	-0.02	-0.83
TP	0.37	-0.83
Ca2+	0.75	0.40
K+	0.93	-0.20
Mg2+	0.54	0.73
Na2+	0.92	0.13
Si	0.96	0.02
Cl-	-0.84	-0.17
SO2	-0.12	-0.14
