# Supplementary material for: The underestimated role of temperature–oxygen relationship in large‐scale studies on size‐to‐temperature response
Source: Ecol Evol. 2017 Aug 11;7(18):7434–41. doi: 10.1002/ece3.3263 (PMC5606864; doi:10.1002/ece3.3263)
Supplement: Supplementary file 2 [file ECE3-7-7434-s002.pdf]

Appendix S2. The list of species excluded from our analyses because of their rarity, by removal from the whole dataset or from a particular stream group; a - occurrence restricted to one stream in a whole dataset; b - only one value within a dataset; c - occurrence restricted to one stream within a particular stream group

**species excluded**	**dataset**	**reason**
Achnanthes stolida	whole	a
Pinnularia ignobilis	whole	a
Navicula cryptotenella	whole	a
Frustulia vulgaris	whole	a
Navicula tripunctata	whole	a
Achnanthes minutissima	whole	b
Achnanthes grana	whole	b
Caloneis lauta	stream group 1	c
Epithemia turgida	stream group 1	c
Fragilaria construens	stream group 1	c
Gomphonema clevei	stream group 1	c
Melosira varians	stream group 1	c
Navicula placentula	stream group 1	c
Nitzschia dissipata	stream group 1	c
Nitzschia fonticola	stream group 1	c
Nitzschia palea	stream group 1	c
Nitzschia paleacea	stream group 1	c
Rhopalodia gibba	stream group 1	c

Amphora inariensis	stream group 2	c
Amphora pediculus	stream group 2	c
Cocconeis pediculus	stream group 2	c
Cymbella sinuata	stream group 2	c
Epithemia sorex	stream group 2	c
Fragilaria arcus	stream group 2	c
Fragilaria construens	stream group 2	c
Gomphonema clavatum	stream group 2	c
Gomphonema clevei	stream group 2	c
Navicula atomus	stream group 2	c
Nitzschia fonticola	stream group 2	c
Nitzschia inconspicua	stream group 2	c
Rhoicosphenia abbreviata	stream group 2	c
Caloneis lauta	stream group 3	c
Cymbella sinuata	stream group 3	c
Diatoma mesodon	stream group 3	c
Fragilaria arcus	stream group 3	c
Fragilaria pinnata	stream group 3	c
Gomphonema clavatum	stream group 3	c
Meridion circulare	stream group 3	c
Navicula placentula	stream group 3	c
