# Supplementary material for: The underestimated role of temperature–oxygen relationship in large‐scale studies on size‐to‐temperature response
Source: Ecol Evol. 2017 Aug 11;7(18):7434–41. doi: 10.1002/ece3.3263 (PMC5606864; doi:10.1002/ece3.3263)
Supplement: Supplementary file 3 [file ECE3-7-7434-s003.pdf]

	Temp	pH	Cond	DOC	DO	NH4	NO3	TN	PO4	TP	Ca	K	Mg	Na	Si	Cl	SO4
**Temp**		0.5934	0.3206	0.186	-0.9225	-0.155	0.5668	-0.5208	0.1922	0.5421	0.403	0.7743	-0.1051	0.2099	0.7598	-0.4383	0.1883
		p=.025	p=.264	p=.524	p=.000003	p=.597	p=.035	p=.056	p=.510	p=.045	p=.153	p=.001	p=.721	p=.471	p=.002	p=.117	p=.519
**pH**	0.5934		0.5347	0.0505	-0.5859	-0.2444	0.3407	-0.0874	0.155	0.4648	0.6717	0.6986	0.2132	0.434	0.7026	-0.7188	0.0375
	p=.025		p=.049	p=.864	p=.028	p=.400	p=.233	p=.766	p=.597	p=.094	p=.009	p=.005	p=.464	p=.121	p=.005	p=.004	p=.899
**Cond**	0.3206	0.5347		0.403	-0.3237	-0.0615	-0.1334	0.1674	-0.2305	0.0535	0.6358	0.7211	0.8959	0.9659	0.825	-0.7972	-0.3041
	p=.264	p=.049		p=.153	p=.259	p=.835	p=.649	p=.567	p=.428	p=.856	p=.015	p=.004	p=.00002	p<.000001	p=.0003	p=.001	p=.291
**DOC**	0.186	0.0505	0.403		-0.1988	-0.2137	0.1572	0.4026	-0.5932	-0.5107	0.2951	0.1688	0.386	0.3121	0.3629	-0.238	-0.2115
	p=.524	p=.864	p=.153		p=.496	p=.463	p=.592	p=.153	p=.025	p=.062	p=.306	p=.564	p=.173	p=.277	p=.202	p=.413	p=.468
**DO**	-0.9225	-0.5859	-0.3237	-0.1988		0.1444	-0.5823	0.3911	-0.2147	-0.4841	-0.3691	-0.7805	0.0739	-0.2277	-0.7491	0.4483	-0.1008
	p=.000003	p=.028	p=.259	p=.496		p=.622	p=.029	p=.167	p=.461	p=.079	p=.194	p=.001	p=.802	p=.434	p=.002	p=.108	p=.732
**NH4**	-0.155	-0.2444	-0.0615	-0.2137	0.1444		-0.3513	0.3914	-0.2747	-0.231	0.1929	-0.1544	0.0425	-0.1539	-0.0551	0.2399	0.4432
	p=.597	p=.400	p=.835	p=.463	p=.622		p=.218	p=.166	p=.342	p=.427	p=.509	p=.598	p=.885	p=.599	p=.851	p=.409	p=.112
**NO3**	0.5668	0.3407	-0.1334	0.1572	-0.5823	-0.3513		-0.2803	0.0005	0.1147	0.0637	0.2572	-0.3769	-0.2185	0.2403	-0.0071	0.1018
	p=.035	p=.233	p=.649	p=.592	p=.029	p=.218		p=.332	p=.999	p=.696	p=.829	p=.375	p=.184	p=.453	p=.408	p=.981	p=.729
**TN**	-0.5208	-0.0874	0.1674	0.4026	0.3911	0.3914	-0.2803		-0.654	-0.7656	0.4089	-0.3551	0.3939	0.0618	-0.138	-0.0711	0.1452
	p=.056	p=.766	p=.567	p=.153	p=.167	p=.166	p=.332		p=.011	p=.001	p=.147	p=.213	p=.163	p=.834	p=.638	p=.809	p=.620
**PO4**	0.1922	0.155	-0.2305	-0.5932	-0.2147	-0.2747	0.0005	-0.654		0.8727	-0.389	0.1188	-0.3711	-0.1205	-0.0605	0.0268	-0.1897
	p=.510	p=.597	p=.428	p=.025	p=.461	p=.342	p=.999	p=.011		p=.00005	p=.169	p=.686	p=.191	p=.681	p=.837	p=.928	p=.516
**TP**	0.5421	0.4648	0.0535	-0.5107	-0.4841	-0.231	0.1147	-0.7656	0.8727		-0.0789	0.5032	-0.2248	0.1129	0.3378	-0.2512	-0.0912
	p=.045	p=.094	p=.856	p=.062	p=.079	p=.427	p=.696	p=.001	p=.00005		p=.789	p=.067	p=.440	p=.701	p=.238	p=.386	p=.757
**Ca**	0.403	0.6717	0.6358	0.2951	-0.3691	0.1929	0.0637	0.4089	-0.389	-0.0789		0.4893	0.4222	0.4401	0.6363	-0.7539	0.4553
	p=.153	p=.009	p=.015	p=.306	p=.194	p=.509	p=.829	p=.147	p=.169	p=.789		p=.076	p=.133	p=.115	p=.014	p=.002	p=.102
**K**	0.7743	0.6986	0.7211	0.1688	-0.7805	-0.1544	0.2572	-0.3551	0.1188	0.5032	0.4893		0.3932	0.6898	0.9318	-0.7181	-0.1545
	p=.001	p=.005	p=.004	p=.564	p=.001	p=.598	p=.375	p=.213	p=.686	p=.067	p=.076		p=.164	p=.006	p=.000001	p=.004	p=.598
**Mg**	-0.1051	0.2132	0.8959	0.386	0.0739	0.0425	-0.3769	0.3939	-0.3711	-0.2248	0.4222	0.3932		0.9157	0.5314	-0.5679	-0.4446
	p=.721	p=.464	p=.00002	p=.173	p=.802	p=.885	p=.184	p=.163	p=.191	p=.440	p=.133	p=.164		p=.000004	p=.051	p=.034	p=.111
**Na**	0.2099	0.434	0.9659	0.3121	-0.2277	-0.1539	-0.2185	0.0618	-0.1205	0.1129	0.4401	0.6898	0.9157		0.7454	-0.7252	-0.4834
	p=.471	p=.121	p<.000001	p=.277	p=.434	p=.599	p=.453	p=.834	p=.681	p=.701	p=.115	p=.006	p=.000004		p=.002	p=.003	p=.080
**Si**	0.7598	0.7026	0.825	0.3629	-0.7491	-0.0551	0.2403	-0.138	-0.0605	0.3378	0.6363	0.9318	0.5314	0.7454		-0.7431	-0.1134
	p=.002	p=.005	p=.0003	p=.202	p=.002	p=.851	p=.408	p=.638	p=.837	p=.238	p=.014	p=.000001	p=.051	p=.002		p=.002	p=.699
**Cl**	-0.4383	-0.7188	-0.7972	-0.238	0.4483	0.2399	-0.0071	-0.0711	0.0268	-0.2512	-0.7539	-0.7181	-0.5679	-0.7252	-0.7431		-0.0551
	p=.117	p=.004	p=.001	p=.413	p=.108	p=.409	p=.981	p=.809	p=.928	p=.386	p=.002	p=.004	p=.034	p=.003	p=.002		p=.852

**SO4**	0.1883<br>p=.519	0.0375<br>p=.899	-0.3041<br>p=.291	-0.2115<br>p=.468	-0.1008<br>p=.732	0.4432<br>p=.112	0.1018<br>p=.729	0.1452<br>p=.620	-0.1897<br>p=.516	-0.0912<br>p=.757	0.4553<br>p=.102	-0.1545<br>p=.598	-0.4446<br>p=.111	-0.4834<br>p=.080	-0.1134<br>p=.699	-0.0551<br>p=.852	
